# Generation of Rat Monoclonal Antibody to Detect Hydrogen Sulfide and Polysulfides in Biological Samples

**DOI:** 10.3390/antiox9111160

**Published:** 2020-11-21

**Authors:** Shingo Kasamatsu, Yuki Kakihana, Taisei Koga, Hisashi Yoshioka, Hideshi Ihara

**Affiliations:** Department of Biological Science, Graduate School of Science, Osaka Prefecture University, Osaka 599-8531, Japan; kasamatsu@b.s.osakafu-u.ac.jp (S.K.); swc04032@edu.osakafu-u.ac.jp (Y.K.); sac04040@edu.osakafu-u.ac.jp (T.K.); hisyoshi.134@gmail.com (H.Y.)

**Keywords:** hydrogen sulfide, persulfides, polysulfides, *N*-ethylmaleimide, monoclonal antibody

## Abstract

Hydrogen sulfide (H_2_S) is endogenously produced by enzymes and via reactive persulfide/polysulfide degradation; it participates in a variety of biological processes under physiological and pathological conditions. H_2_S levels in biological fluids, such as plasma and serum, are correlated with the severity of various diseases. Therefore, development of a simple and selective H_2_S measurement method would be advantageous. This study aimed to generate antibodies specifically recognizing H_2_S derivatives and develop a colorimetric immunoassay for measuring H_2_S in biological samples. We used *N*-ethylmaleimide (NEM) as an H_2_S detection agent that forms a stable bis-S-adduct (NEM-S-NEM). We also prepared bis-S-heteroadduct with 3-maleimidopropionic acid, which, in conjugation with bovine serum albumin, was to immunize Japanese white rabbits and Wistar rats to enable generation of polyclonal and monoclonal antibodies, respectively. The generated antibodies were evaluated by competitive enzyme-linked immunosorbent assay. We could obtain two stable hybridoma cell lines producing monoclonal antibodies specific for NEM-S-NEM. By immunoassay with the monoclonal antibody, the H_2_S level in mouse plasma was determined as 0.2 μM, which was identical to the level detected by mass spectrometry. Taken together, these monoclonal antibodies can be a useful tool for a simple and highly selective immunoassay to detect H_2_S in biological samples.

## 1. Introduction

Hydrogen sulfide (H_2_S), which was first documented as a highly hazardous chemical with a distinct smell of a rotten egg roughly 300 years ago, is a well-established gaseous molecule with numerous biological activities [1]. To date, studies have reported H_2_S participation in a variety of biological processes, including antioxidative stress response [2,3], protection against ischemia-reperfusion injury [4], and the development/progression of neurodegenerative diseases [5], under physiological and pathological conditions. It has been reported that H_2_S is endogenously generated in mammalian cells and tissues from cysteine or cysteine derivatives via reactions catalyzed by cystathionine β-synthase, cystathionine γ-lyase, and 3-mercaptopyruvate sulfurtransferase [1,6,7], as well as by degradation of persulfides/polysulfides [8,9,10]. H_2_S has been shown to function as a gaseous signaling compound [1] with cytoprotective capabilities. For example, Kakinohana et al. reported that inhaling H_2_S at 80 ppm after ischemia/reperfusion could prevent motor neuron degeneration in the ventral horn of the lumbar spinal cord via persulfidation of nuclear factor-κB (NF-κB) and p65 [11]. Indeed, H_2_S level in biological fluids, such as plasma and serum, is correlated with the severity of various diseases [12,13,14,15,16]. However, the chemical mechanisms associated with the biological activities of H_2_S are not fully elucidated.

H_2_S is soluble in water and hence in biological fluids, such as plasma and extracellular matrix. The percentage composition of H_2_S in biological fluids depends on temperature and pH. Approximately 20% and 80% of H_2_S exist in the gaseous and deprotonated (hydrogen sulfide anion, HS^−^) forms, respectively, while a very negligible amount is found as sulfide anions (S^2−^), in a physiological environment (pH 7.4, 37 °C). However, 40% of H_2_S exists in the gaseous form at pH 7.4 and 25 °C [17]. Several methods are available for H_2_S detection, from the classic colorimetric assay using methylene blue [18,19] to the more recent techniques, such as gas chromatography [20], high-performance liquid chromatography (HPLC) [21], and mass spectrometry (MS) [22]. Fluorescent, electrochemical, and chemiluminescent methods with multiple chemical probes to visualize intracellular H_2_S levels in living cells have also been developed [23]. In these methods, H_2_S is often derivatized to form bis-S-adduct using several alkylating agents, such as monobromobimane (MBB), β-(4-hydroxyphenyl)ethyl iodoacetamide (HPE-IAM), and maleimide-based compounds, including *N*-ethylmaleimide (NEM) and *N*-(1-pyrene) maleimide [14,16,17]. Although these recently developed techniques are highly specific and sensitive with much lower detection limits, they require costly instruments, such as a mass spectrometer.

From the viewpoint of disease prevention and diagnosis, it is important to evaluate H_2_S levels in biological fluids, and a simple and highly selective H_2_S measurement method is required. Colorimetric immunological detection methods, such as enzyme-linked immunosorbent assay (ELISA), are easy to handle, widely used, and require a microplate reader, which is a relatively low-cost piece of equipment compared with other pieces of equipment used in recently developed methods, such as gaseous or liquid chromatographs and mass spectrometers. Therefore, the present study aimed to generate a specific monoclonal antibody to detect H_2_S using NEM as the H_2_S detection agent, by immunizing rabbit and rat with a protein conjugated with bis-S-adduct of NEM (NEM-S-NEM) as immunogen, and to develop a simple colorimetric immunoassay for detection of H_2_S in biological samples.

## 2. Materials and Methods

### 2.1. Materials

NEM and bovine serum albumin (BSA) were obtained from Nacalai Tesque (Kyoto, Japan). Furthermore, 3-maleimidopropionic acid (MPA) and water soluble carbodiimide (WSC) were obtained from Tokyo Chemical Industry (Tokyo, Japan). All other chemicals and reagents were procured from common suppliers and were of the highest grade commercially available.

### 2.2. Preparation of NEM-S-Adducts

To prepare NEM-S-NEM and NEM-S-MPA, a bis-S-heteroadduct of NEM and MPA, 50 mM NEM was reacted with 50 mM sodium hydrosulfide (NaHS) in 500 mM sodium phosphate buffer (pH 7.4) containing 50% methanol with or without 50 mM MPA with continuous stirring at room temperature (25–28 °C) for 1 h. The NEM-S-NEM and NEM-S-MPA adducts were purified by HPLC (JASCO Corporation, Tokyo, Japan) using a C18 reverse-phase column (CAPCELL PAK C18 UG80 20 × 250 mm; Shiseido, Tokyo, Japan) and a linear gradient of solvent A (water containing 0.1% formic acid [FA]) and solvent B (100% methanol) (0% B at 0 min; 60% B at 70 min) at a flow rate of 6 mL/min. The elution was monitored by detecting absorbance at 220 nm. The purified NEM-S-NEM and NEM-S-MPA were confirmed by HPLC with a C18 reverse-phase column (Mightysil RP-18 GP 3.0 × 75 mm; Kanto Chemical, Tokyo, Japan) using a linear gradient of solvent A (water containing 0.1% FA) and solvent B (100% methanol) (0% B at 0 min; 70% B at 10 min) at a flow rate of 1 mL/min.

To produce NEM-adduct of cysteine (NEM-Cys), which was used as a competitor in competitive ELISA, 500 mM NEM was incubated with 50 mM cysteine in 100 mM sodium phosphate buffer (pH 7.4) containing 50% methanol with continuous stirring at room temperature (25–28 °C) for 1 h. *N*-acetyl cysteine (NAC) was also incubated with NEM to generate NEM-NAC that was used to prepare an affinity column for removal of putative contaminants from the rabbit antiserum, which included antibodies recognizing NEM-conjugated thiols, such as cysteine, glutathione, and protein cysteine residues. The produced NEM-Cys and NEM-NAC were purified by HPLC as described above. The purified NEM-NAC was conjugated to TOYOPEARL AF-Amino-650M beads (particle size, 65 μm; Tosoh, Kyoto, Japan) according to the manufacturer’s instructions.

**Figure 1:**
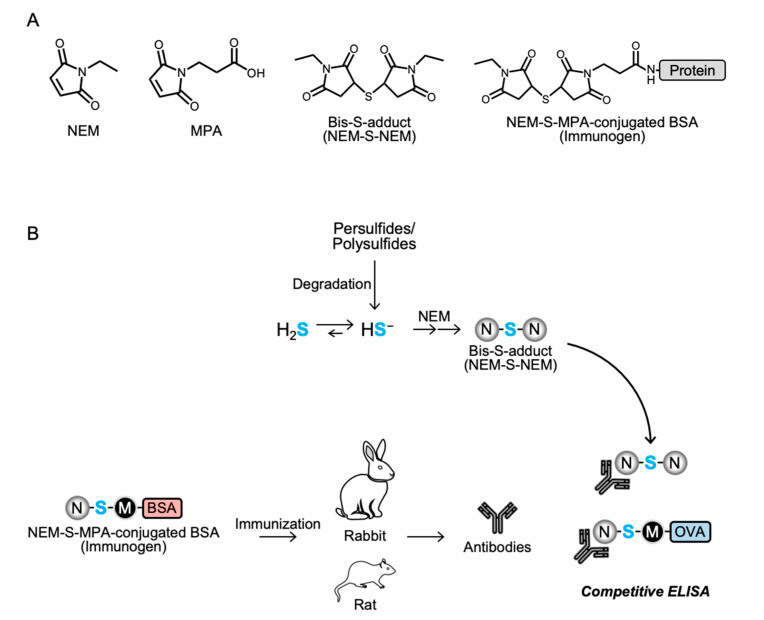
Schematic representation of the reaction of hydrogen sulfide with *N*-ethylmaleimide. (**A**) Molecular structures of chemicals used in this study. (**B**) H_2_S exists as approximately 20% as the gaseous form (H_2_S), 80% as the deprotonated form (hydrogen sulfide anion, HS^−^) in a physiological environment. *N*-ethylmaleimide (NEM) reacts with HS^−^ to form an intermediate S-adduct, and a subsequent reaction with NEM results in the formation of a stable bis-S-adduct, NEM-S-NEM. NEM-S-NEM can also be formed via decomposition of persulfides/polysulfides. MPA, 3-maleimidopropionic acid; BSA, bovine serum albumin; OVA, ovalbumin.

The chemical structures of NEM-S-adducts were characterized by liquid chromatography-electrospray ionization-tandem MS (LC-ESI-MS/MS), which was performed on a triple quadrupole mass spectrometer (Xevo TQD; Waters, Milford, MA) coupled with an Alliance e2695 system (Waters). NEM-S-adducts were separated in an Alliance e2695 system using a C18 reverse-phase column (Mightysil RP-18 GP 2.0 × 50 mm; Kanto Chemical) with a linear gradient of solvent A (water containing 0.1% FA) and solvent B (100% methanol) (1% B at 0 min; 99% B at 5 min) at a flow rate of 0.3 mL/min. The mass spectrometer was operated in positive mode under the following conditions: capillary voltage, 1000 V; desolvation gas, nitrogen, at 1000 L/h at 500 °C. NEM-S-adducts were identified by multiple reaction monitoring (MRM). The MRM parameters are provided in Supplementary Table S1. Chemical structures of the NEM-adducts are depicted in Figure 1A and Supplementary Figures S1 and S2.

### 2.3. Conjugation of NEM-S-MPA to Protein

To prepare an immunogen, NEM-S-MPA was conjugated to BSA via a coupling reaction of the propionic acid moiety of NEM-S-MPA and the primary amine moiety (lysine residue) of BSA using WSC. In brief, NEM-S-MPA was incubated with 3.3 mg/mL BSA and 30 mM WSC in 0.1 M 2-(*N*-morpholino)ethanesulfonic acid buffer (pH 5.5) with continuous stirring at room temperature (25–28 °C) for 12 h. After centrifugation at 750× *g*, 4 °C for 5 min, the resultant supernatant was transferred into a desalting column (PD-10, GE Healthcare, Little Chalfont, England), pre-equilibrated with ice-cold phospho-buffered saline (PBS), to remove free WSC and NEM-S-MPA. Protein fractions were collected with ice-cold PBS, and the protein concentration was determined by the Bradford method with BSA as a standard. To prepare an antigen for ELISA, ovalbumin (OVA; CalbioChem, San Diego, CA, USA) was used instead of BSA.

To verify NEM-S-MPA conjugation onto BSA and OVA proteins, NEM-S-MPA-conjugated BSA and OVA proteins were digested by chymotrypsin and the generated NEM-S-MPA-containing peptide fragments were detected by LC-ESI-MS. Chymotryptic digestion of NEM-S-MPA-conjugated BSA and OVA proteins was carried out by the general protocol. In brief, the protein was denatured in 100 mM Tris-HCl (pH 8.0) containing 6 M urea at 37 °C for 1 h, reduced in the presence of 20 mM dithiothreitol at 37 °C for 30 min, and then incubated with 50 mM iodoacetamide at 37 °C for 30 min. The reaction mixture was diluted 1.6 times with 100 mM Tris-HCl (pH 8.0) containing 10 mM CaCl_2_ and 6.25 μg/mL chymotrypsin, and further incubated at 37 °C for 12 h. The chymotryptic digest was enriched by a C18 column (Discovery^®^ DSC-18 100 mg, Sigma-Aldrich, St. Louis, MO, USA), and the eluate was concentrated *in vacuo*, and then the NEM-S-MPA-containing peptide fragments in the solution were analyzed by LC-ESI-MS analysis. LC-ESI-MS analysis was performed by the same LC-MS system described above. Peptide fragments were separated in an Alliance e2695 system using a C18 reverse-phase column (Mightysil RP-18 GP 2.0 × 50 mm) with a linear gradient of solvent A (water containing 0.1% FA) and solvent B (100% methanol) (1% B at 0 min; 99% B at 10 min) at a flow rate of 0.3 mL/min. The MS setting parameters were the same as described above. NEM-S-MPA-containing peptide fragments, the predicted mass of which was calculated by ProteinProspector (version 6.2.2) developed by the University of California San Francisco Mass Spectrometry Faculty (http://prospector.ucsf.edu), were identified by selected ion monitoring (SIM). The SIM parameters are provided in Supplementary Table S2.

### 2.4. Preparation of Anti-NEM-S-NEM Antibody

This study was performed in accordance with the Guidelines for Animal Experimentation of Osaka Prefecture University, Osaka, Japan. All animal experiments were approved by the Animal Ethical Committee of Osaka Prefecture University. To obtain the anti-NEM-S-NEM rabbit polyclonal antibody (pAb), a 10 week-old male Japanese white rabbit (Kiwa Laboratory Animals, Wakayama, Japan) was immunized by subcutaneous administration of NEM-S-MPA-conjugated BSA with Freund’s complete adjuvant (Difco Laboratories, Detroit, MI, USA). Booster doses of the same immunogen plus Freund’s incomplete adjuvant were administered five times every 2 weeks. Three days after the last booster injection, the animal was anesthetized with isoflurane and sacrificed by collection of whole blood volume followed by serum separation. To eliminate putative contamination with antibodies recognizing NEM-conjugated thiols, including cysteine, glutathione, and protein cysteine residues, in the rabbit antiserum, the serum was subjected into an NEM-NAC-conjugated TOYOPEARL AF-Amino-650 M beads affinity column, and the flow-through fraction, collected in a new tube, was used as rabbit pAb. Antibody specificity was confirmed by competitive ELISA using NEM, NEM-Cys, and NEM-S-NEM (detailed procedure is described in Section 2.5).

The rat monoclonal antibody (mAb) specific for NEM-S-NEM was prepared as described previously [24], with slight modifications. A murine myeloma cell line (P3X63-Ag653) was used for cell fusion. In brief, a 10 week-old male Wister rat (Kiwa Laboratory Animals) was subcutaneously administrated NEM-S-MPA-conjugated BSA plus Freund’s complete adjuvant. Booster doses of the same immunogen plus Freund’s incomplete adjuvant were administered three times every 2 weeks. Three days after the last booster dose, the immunized rat was anesthetized with isoflurane and sacrificed by collection of whole blood volume, and the spleen tissue was harvested. After washing the spleen cells twice with ice-cold Dulbecco’s modified Eagle’s medium (DMEM, Wako Pure Chemical, Osaka, Japan) without serum, cells were mixed with the myeloma cells at a ratio of 4:1. After centrifugation, the resultant supernatant was removed, and the cell pellets were resuspended in DMEM containing 50% (*w*/*v*) polyethylene glycol 4000 (Merck Millipore, Darmstadt, Germany), which was added dropwise. After washing cells with DMEM containing 15% fetal bovine serum (FBS, Cytosystems, Castle Hill, NSW, Australia), cells were resuspended in GIT medium (Wako Pure Chemical) supplemented with 10% FBS, 1% penicillin-streptomycin, and 1× HAT media supplement (Sigma-Aldrich, St. Louis), and then seeded into four 96-well plates (Iwaki, Tokyo, Japan). On day 7 after cell fusion, the culture supernatant of the growing hybridoma cells was tested by competitive ELISA, and positive hybridoma clones were screened. Twenty-four positive hybridoma clones were successfully obtained. Finally, two stable hybridoma cell lines (1C6 and 2D7) producing anti-NEM-S-NEM mAb were selected and used for further studies.

### 2.5. Competitive ELISA

Specificity of the rabbit and rat antisera was examined by competitive ELISA; the culture supernatants of hybridoma cells were also screened for production of anti-NEM-S-NEM rat mAb by competitive ELISA. In brief, ELISA plates (Iwaki) were coated with NEM-S-MPA-conjugated OVA (0.2 μg/well in PBS) at 4 °C overnight. The plates were washed three times with Tris-buffered saline containing 0.05% Tween 20 (TBST) and then blocked with 5 mg/mL gelatin for 30 min at 37 °C. The plates were washed three times with TBST, and a mixture of antibodies (the rabbit/rat antisera, rabbit pAb, or culture supernatant of hybridoma cells) with 2-fold dilution series of competitors (NEM, NEM-Cys, and NEM-S-NEM; 0.24 μM–1 mM) pre-incubated at 37 °C for 30 min was added to each well. After 30 min incubation at 37 °C, the plates were washed three times with TBST, and horse radish peroxidase-conjugated anti-rabbit immunoglobulin g (IgG) or anti-rat IgG antibody (1:5000 dilution; Santa Cruz Biotechnology, Dallas, TX, USA) was added to each well. After 30 min incubation at 37 °C, the plates were washed three times with TBST followed by addition of 0.5 mg/mL *o*-phenylenediamine (Nacalai Tesque) in 0.1 M citrate-phosphate buffer (pH 5.0) containing 0.024% hydrogen peroxide to each well and further incubated at room temperature (25–28 °C) for 30 min. The colorimetric reaction was terminated by the addition of 2 M H_2_SO_4_. Absorbance was measured at 490 nm using a microplate reader (Bio-Rad model 450; Hercules, CA, USA).

In a model experiment for biological samples, a reaction mixture containing 1 mM NEM and various concentrations of NaHS (6.3, 25, and 100 μM), incubated in PBS at 37 °C for 1 h, was used as a competitor for competitive ELISA with the rat mAb (1C6).

To evaluate whether the immunoassay was useful for analyzing biological samples, mouse plasma obtained from 20-week-old male C57BL/6J mice (Kiwa Laboratory Animals) was utilized. The mice (*n* = 5) were sacrificed by exsanguination under general anesthesia; plasma was prepared, flash frozen in liquid nitrogen, and stored at −80 °C until further procedures. Plasma was reacted with 10 mM NEM in PBS containing 50% methanol at 37 °C for 1 h. After centrifugation, the supernatant was collected, dried *in vacuo*, and then dissolved in 0.1% FA, an aliquot of which was analyzed by LC-ESI-MS/MS for quantification of the formed NEM-S-NEM as described previously [8]. For detecting the formed NEM-S-NEM by competitive ELISA to enrich the formed NEM-S-NEM, samples were loaded onto a C18 column (Discovery^®^ DSC-18 500 mg, Sigma-Aldrich), the column was washed with 0.1% FA containing 25% methanol, then NEM-S-NEM was collected with 0.1% FA containing 50% methanol. Eluates were dried in vacuo, and then dissolved in PBS. The final solutions, 67-fold concentrated compared with the original plasma, were utilized as competitors for competitive ELISA as described above. Mouse plasma samples were mixed with rat mAb at a ratio of 1:4. The concentration in the original plasma was calculated by the following formula: the original concentration = the concentration determined by competitive ELISA × 5 (dilution factor) ÷ 67 (concentration factor). The exact values of original mouse plasma determined by competitive ELISA and LC-ESI-MS/MS analysis are described in Supplementary Table S3.

### 2.6. Statistical Analyses

Data are presented as mean ± standard error of the mean (SEM) of at least three independent experiments unless otherwise specified. For statistical comparisons, we utilized two-way analysis of variance (ANOVA) followed by Tukey’s multiple comparisons test. All analyses were performed using GraphPad Prism software (GraphPad Software, La Jolla, CA, USA). *p* < 0.05 was considered statistically significant.

## 3. Results

### 3.1. Strategy to Generate Antibodies for Detection of H_2_S

The strategy to generate antibodies for detection of H_2_S is illustrated in Figure 1B. H_2_S was derivatized with NEM to form a stable bis-S-adduct, NEM-S-NEM. NEM-S-MPA was synthetized and conjugated to BSA and OVA and used to immunize rabbits and rats to generate polyclonal and monoclonal antibodies, respectively. The generated antibodies were evaluated by competitive ELISA.

### 3.2. Preparation of NEM-S-MPA, Immunogen, and Antigen

**Figure 2:**
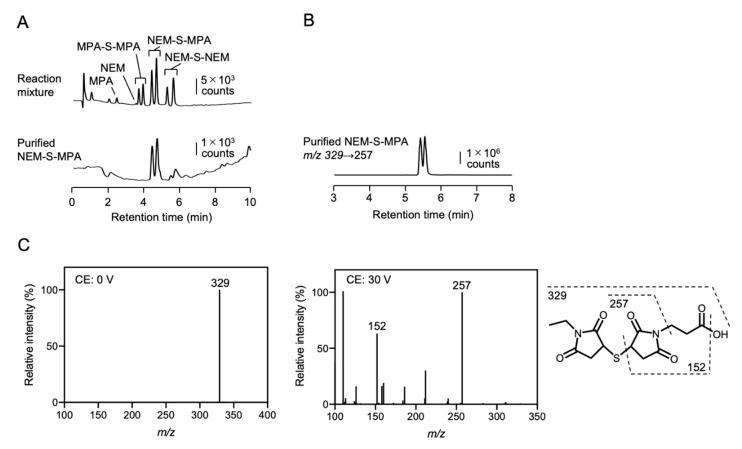
Preparation of NEM-S-adducts. NEM-S-adducts were prepared by a reaction of NEM and 3-maleimidopropionic acid (MPA) in the presence of sodium hydrosulfide (NaHS). (**A**) Representative HPLC chromatograms of the reaction mixture (*upper*) and a purified bis-S-heteroadduct of NEM and MPA, NEM-S-MPA (*lower*). (**B**) Representative liquid chromatography-electrospray ionization-tandem MS (LC-ESI-MS/MS) chromatogram of the purified NEM-S-MPA. (**C**) Mass spectra of non-fragment ion (*left*), fragment ions (*center*) and assigned chemical structures (*right*) indicating cleavage sites by dashed lines of purified NEM-S-MPA.

To generate a bis-S-heteroadduct of NEM and MPA, NEM-S-MPA, which can conjugate to proteins via a coupling reaction between the propionic acid moiety of MPA and the amino moiety of protein lysine residues, NEM was reacted with NaHS in the presence of MPA. As shown in Figure 2A (*upper*), the formed NEM-S-MPA and bis-S-adducts of NEM and MPA, NEM-S-NEM and MPA-S-MPA, respectively, were detected by HPLC. Several studies have reported that the reaction of sulfide with maleimides, such as NEM, can form two distinct diastereomers because the sulfur can react with either C3 or C4 of the maleimide ring [25,26,27]. In fact, the formed respective bis-S-adducts were observed as double peaks by HPLC analysis (Figure 2A, *upper*). Although the reaction mixture contained NEM-S-NEM, MPA-S-MPA, free MPA, and NEM, the formed NEM-S-MPA were easily separated from the other adducts by HPLC because they have different hydrophobicity (Figure 2A, *lower*). The purified NEM-S-MPA was also detected as double peaks at retention times of 4.4 and 4.7 min, which were identical to that of the formed NEM-S-MPA in the reaction mixture. By LC-ESI-MS/MS analysis, the purified NEM-S-MPA was detected as protonated molecules of *m*/*z* 329 and the MS chromatogram indicated double peaks (Figure 2B). The product ion assignments also evidenced that NEM-S-MPA was successfully prepared (Figure 2C).

**Figure 3:**
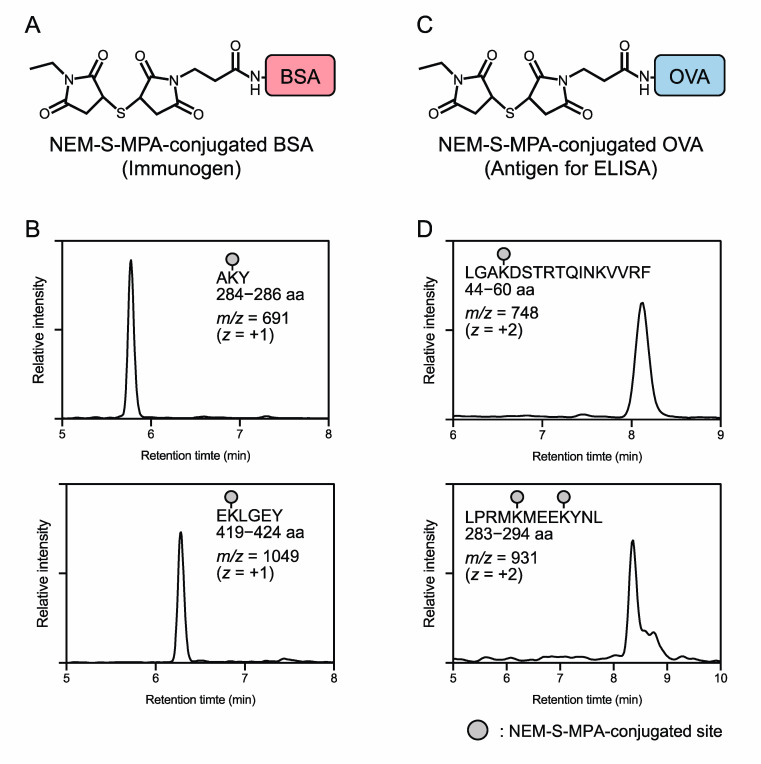
Preparation of immunogen and antigen. Immunogen (**A**) and antigen for ELISA (**C**) were prepared by a conjugation of NEM-S-MPA to bovine serum albumin (BSA) and ovalbumin (OVA), respectively. Representative MS chromatograms of peptide fragments containing NEM-S-MPA modifications derived from NEM-S-MPA-conjugated BSA (**B**) and OVA (**D**).

To generate the immunogen and antigen, BSA or OVA was incubated with WSC in the presence or absence of the purified NEM-S-MPA, forming NEM-S-MPA-conjugated BSA or OVA, respectively. To confirm the conjugation of NEM-S-MPA to BSA and OVA proteins, the proteins were digested by chymotrypsin and subjected to LC-ESI-MS analysis. As shown in Figure 3 and Supplementary Figures S3 and S4, the MS analysis detected several peptide fragments containing NEM-S-MPA obtained from NEM-S-MPA-conjugated BSA and OVA proteins, respectively. A certain amount of precipitates was observed after the reaction of WSC and the proteins, both in the absence or presence of NEM-S-MPA, which suggests that fully crosslinked proteins might be denatured and aggregated. After removal of the precipitates by centrifugation, the resultant supernatant was subjected to MS analysis for checking whether crosslinkages were successfully formed for use as an immunogen and antigen for ELISA. These data indicated successful conjugation of NEM-S-MPA to BSA or OVA.

### 3.3. Generation of Rabbit pAb

**Figure 4:**
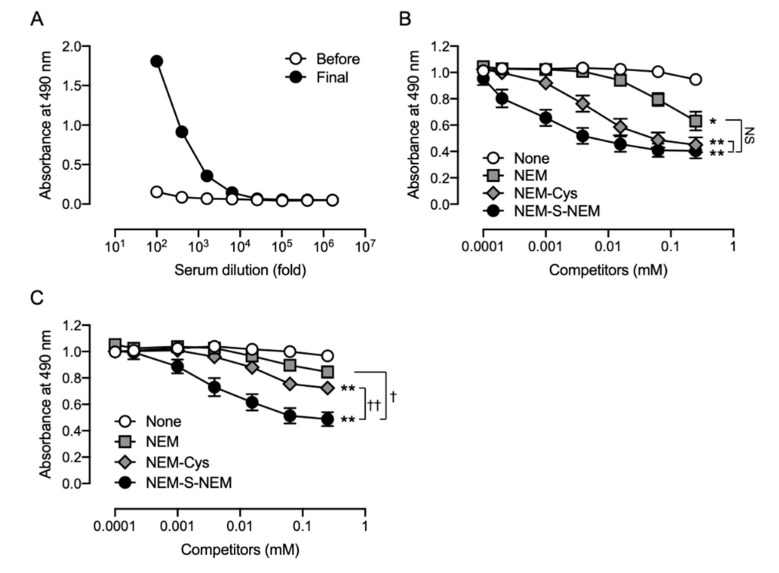
Characterization of rabbit polyclonal antibody. Japanese white rabbit was subcutaneously injected with NEM-S-MPA-conjugated BSA. The antibody titer in rabbit antiserum obtained from the immunized rabbit before the immunization (*before*) and 3 days after the last booster injection (*final*) was measured by ELISA with NEM-S-MPA-conjugated OVA as an antigen (**A**). Antibody specificity of the rabbit antiserum (**B**) and the flow-through (rabbit polyclonal antibody (pAb)) (**C**), which was obtained by applying the antiserum onto an affinity column to remove putative contamination of antibodies to recognize NEM-labeled thiols, was confirmed by a competitive ELISA using NEM-S-NEM, NEM-labeled cysteine (NEM-Cys), and NEM as competitors. NEM-Cys, NEM-labeled cysteine. * *p* < 0.001, ** *p* < 0.0001 vs none; ^†^ *p* < 0.001, ^††^ *p* < 0.0001 vs. NEM-S-NEM, compared by Two-way ANOVA with Tukey’s multiple comparisons test.

After immunization of the rabbit with NEM-S-MPA-conjugated BSA, antibody titers against NEM-S-MPA were measured by ELISA using NEM-S-MPA-conjugated OVA as an antigen. As shown in Figure 4A, the antibody titers against NEM-S-MPA in the rabbit serum dramatically increased due to the subcutaneous injection of NEM-S-MPA-conjugated BSA. Competitive ELISA showed that NEM-S-NEM partially inhibited binding of the pAb to NEM-S-MPA-conjugated OVA in a concentration-dependent manner (Figure 4B). There were no significant differences between the raw serum and rabbit pAb, indicating that the pAb recognized, at least in part, NEM-S-NEM. These data indicated that the strategy employed in this study was appropriate for the generation of antibody recognizing NEM-S-NEM.

However, NEM-Cys and NEM also inhibited the binding of the pAb to NEM-S-MPA-conjugated OVA (Figure 4B), indicating that the pAb could also recognize NEM and NEM-conjugated thiols, such as cysteine and glutathione. These antibodies, recognizing NEM-conjugated thiols and NEM, were eliminated from the polyclonal antiserum using an affinity column of NEM-NAC-conjugated TOYOPEARL AF-Amino-650 M beads. The binding property of the flow-through fraction was assessed by competitive ELISA; however, the antibodies recognizing NEM and NEM-conjugated thiols were only partially removed from the antiserum (Figure 4C). We performed a statistical analysis to compare the results of Figure 4B,C. The statistical analysis indicated that the reactivity of the rabbit pAb against NEM-Cys and NEM was significantly lower than those of the rabbit serum, while the sensitivity of the rabbit pAb against NEM-S-NEM was significantly lower than that of the rabbit serum. Therefore, as the antibody specificity was not high enough, we decided to not use the rabbit pAb prepared herein for further experiments.

### 3.4. Generation of Rat Monoclonal Antibodies

**Figure 5:**
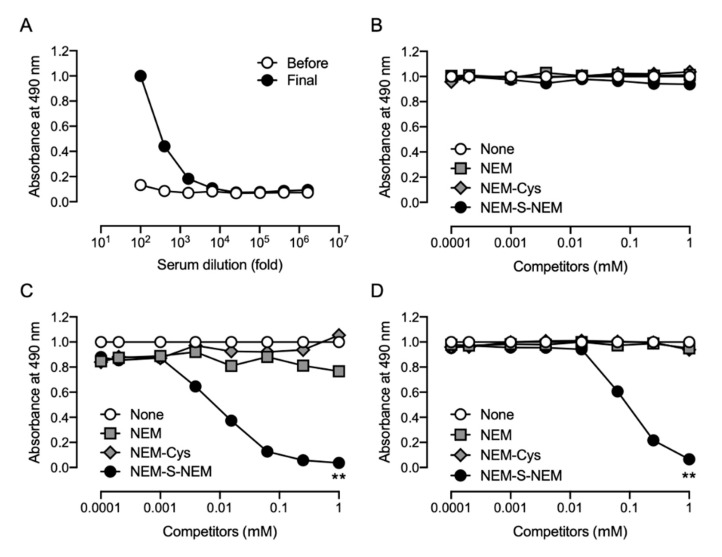
Characterization of rat monoclonal antibody. Wister rat was subcutaneously injected with NEM-S-MPA-conjugated BSA. The antibody titer in rat antiserum obtained from the immunized rat before the immunization (*before*) and 3 days after the last booster injection (*final*) was measured by ELISA with NEM-S-MPA-conjugated OVA as an antigen (**A**). Antibody specificity of the rat antiserum (**B**) and two monoclonal antibodies (mAb) produced by stable hybridoma cell lines (1C6 and 2D7) (**C**,**D**) was confirmed by competitive ELISA using NEM-S-NEM, NEM-Cys, and NEM as competitors. ** *p* < 0.0001 vs. none, compared by two-way ANOVA with Tukey’s multiple comparisons test.

**Figure 6:**
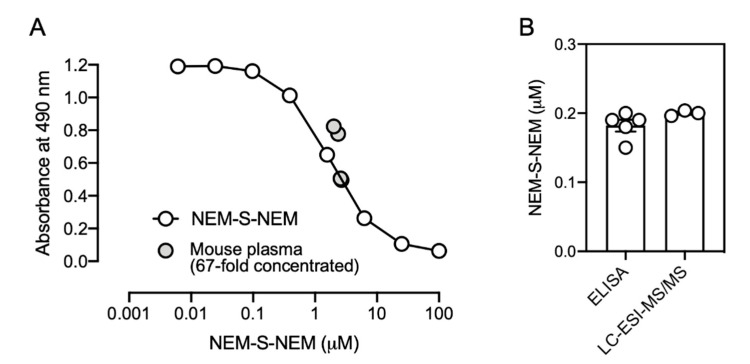
Detection of NEM-S-NEM in mouse plasma by competitive ELISA. (**A**) Mouse plasma was incubated in PBS containing 5 mM NEM and 50% methanol at 37 °C for 1 h, and the formed NEM-S-NEM in the reaction mixture was enriched by a solid phase extraction. The eluates were dried *in vacuo*, dissolved in PBS, then the concentration was determined by competitive ELISA with anti-NEM-S-NEM mAb (clone 1C6). Purified NEM-S-NEM was used as a standard. (**B**) The concentration in the original mouse plasma was calculated by the following formula: the original concentration = the concentration determined by competitive ELISA (2.44 ± 0.11 μM) × 5 (dilution factor) ÷ 67 (concentration factor). The formed NEM-S-NEM in the reaction mixture was also determined by LC-ESI-MS/MS analysis. The exact values in original mouse plasma determined by competitive ELISA and LC-ESI-MS/MS analysis are described in Supplementary Table S3.

The antibody titer in the serum of NEM-S-MPA-conjugated BSA-treated Wister rat was confirmed by competitive ELISA with NEM-S-MPA-conjugated OVA as the antigen (Figure 5A). Moreover, all competitors, including NEM-Cys and NEM-S-NEM, did not compete for immunoreaction with the rat antiserum (Figure 5B). We confirmed that a higher concentration of NEM-S-NEM slightly competed the immunoreactivity of the rat serum against NEM-S-MPA-conjugated OVA (data not shown), indicating that the rat serum contained antibodies against NEM-S-NEM, but the titer was not high enough to detect the bis-S-adduct at a concentration below 1 mM. Further, we successfully generated 24 hybridoma clones, via fusion of immunized rat spleen cells and murine myeloma cell line, producing mAbs that reacted with NEM-S-NEM. After several screening processes, we obtained two hybridoma cell lines (1C6 and 2D7) that produced mAbs with high specificity toward NEM-S-NEM but not NEM-Cys and NEM (Figure 5C,D) as well as NEM-labeled glutathione (data not shown). We performed a model experiment for detection of NEM-S-NEM in a reaction mixture containing different NaHS concentrations (6.3, 25, 100 μM) and excess amount of NEM (1 mM) by competitive ELISA using the anti-NEM-S-NEM rat mAb (1C6). The reaction mixture containing NEM and NaHS, which was pre-incubated at 37 °C for 30 min to form NEM-S-NEM, was used as a competitor. As shown in Supplementary Figure S5, the reaction mixture could compete for immunoreactivity with the immunogen in a NaHS concentration-dependent manner and the competition efficacy was equal to that of purified NEM-S-NEM. To verify whether the immunoassay developed in this study was useful for the detection of H_2_S in biological samples, we tried to measure the H_2_S level in mouse plasma by competitive ELISA. As shown in Figure 6, the H_2_S level in mouse plasma was determined as 0.2 ± 0.01 μM by the immunological method, similar to the results detected by LC-ESI-MS/MS analysis (0.2 ± 0.004 μM). These results indicate that the immunological method developed herein is a highly specific method for detection of NEM-S-NEM with an identical specificity to LC-ESI-MS/MS analysis.

## 4. Discussion

In the present study, we developed a strategy to generate antibodies for detection of H_2_S that involved the following steps: (i) derivatization of H_2_S by NEM, (ii) preparation of NEM-S-MPA-conjugated BSA and OVA, (iii) immunization of animal models and isolation of antibodies. H_2_S is derivatized by several alkylating agents, such as MBB, HPE-IAM, and maleimide-based compounds including NEM, to form a stable bis-S-adduct. In this study, we used NEM to derivatize H_2_S because NEM is a well-known thiol alkylating agent that is cost-effective, easily available, and easy to handle. Furthermore, because several maleimide compounds are commercially available, heterogenic bis-S-adduct analogous to NEM-S-NEM, such as NEM-S-MPA, was successfully generated; this could conjugate protein via a coupling reaction between the propionic acid moiety of MPA and the amino moiety of protein. Next, we prepared a protein-conjugated bis-S-adduct NEM-S-MPA as the immunogen and antigen. Since the structure of NEM-S-MPA is analogous to that of NEM-S-NEM, NEM-S-MPA-conjugated protein was expected to function as an immunogen for the antibodies recognizing NEM-S-NEM. Moreover, we employed two different animal species: rabbit for pAb and rat for mAb generation because it is known that rabbits produce high titers of high-affinity antibodies, even against antigens that are not immunogenic in mice [28,29,30]. The antibody titer in antiserum obtained from both immunized animals markedly increased after the administration of NEM-S-MPA-conjugated BSA as immunogen. Competitive ELISA showed that rabbit antiserum contained antibodies recognizing NEM-S-NEM, indicating that our strategy for generation of NEM-S-NEM-recognizing antibody was appropriate. However, since the rabbit pAb also showed high-affinity to NEM-labeled thiols and NEM, the rabbit pAb was not suitable for the detection of H_2_S in biological samples containing large amounts of thiols, such as cysteine, glutathione, and protein cysteine residue. Further, we fused spleen cells obtained from the immunized rat and murine myeloma cells to generate hybridoma cell lines. After several screening processes, we finally obtained two stable hybridoma cell lines (1C6 and 2D7) producing rat mAbs that were highly specific for NEM-S-NEM but not NEM-Cys and NEM. Furthermore, we stoichiometrically detected NEM-S-NEM in a reaction mixture of NEM-S adducts by competitive ELISA with the generated rat mAb (clone 1C6). Thus, we successfully generated two mAbs highly specific for NEM-S-NEM, the bis-S-adduct of NEM. The mAbs prepared herein may be used to detect derivatized H_2_S, and hence H_2_S levels, in biological fluids, such as plasma, serum, and cerebrospinal fluid.

Early studies have documented the concentration of H_2_S in plasma as 20–80 μM; although recent reports indicate it to be lower (submicromolar), a large labile pool of reactive persulfides/polysulfides exist that may degrade into H_2_S in response to biological stimuli [31]. In fact, it was recently reported that reactive persulfides and polysulfides, such as glutathione persulfide, are endogenously produced at significant levels in various organisms, including mammals [8,32,33]. Persulfides/polysulfides exist not only as low-molecular-weight compounds but also as high-molecular-weight compounds, including proteins [8,32,34]. Accumulating evidence indicates that persulfides/polysulfides play important roles in multiple cellular functions, such as antioxidative stress response, anti-inflammation, regulation of redox signal transduction, and mitochondrial biogenesis, via their unique chemical properties [8,32,35,36]. Polysulfides, which are also considered as bound sulfide, can release sulfides from their bound form in acidic environments [37]. Recently, Ikeda et al. reported a novel combined assay by modifying sulfide antioxidant buffer (SAOB), a strong redox buffer, composed of 0.5 M sodium salicylate, 0.12 M ascorbic acid, and 2.2 M NaOH, to produce an Elimination Method of Sulfide from Polysulfide (EMSP) treatment solution that liberates sulfides [38]. The sulfides released from polysulfides via reduction under alkaline conditions can be measured by H_2_S detection techniques, and hence polysulfides are considered as a pool of H_2_S. Therefore, the concentration of H_2_S in biological samples reported previously might have included degradation products of reactive persulfides/polysulfides. With regard to the yield of NEM-S-NEM formation in biological samples, the derivatization of H_2_S to NEM-S-NEM in the study was carried out by 1 h incubation in PBS containing 5 mM NEM at 37 °C, conditions at which we expected that a section of the sulfides was converted to NEM-S-NEM because some types of polysulfides are relatively stable at neuronal pH. Therefore, it is required to develop a derivatization procedure to degrade polysulfides to sulfides, which can definitely be derivatized by NEM to form NEM-S-NEM. To avoid this and enable precise measurement of H_2_S in biological samples containing persulfides/polysulfides, it is necessary to carefully control the pH and temperature of the experimental conditions.

Our previous studies with LC-ESI-MS/MS analysis revealed that oxidized glutathione tetrasulfide, a biological polysulfide, can be decomposed by an electrophilic reaction with alkylating compounds, such as iodoacetamide, MBB, and NEM, especially at a higher pH (pH 8.0) [8,9,10]. Furthermore, we recently established that polysulfides can be degraded by incubating them with reductants, such as dithiothreitol, at alkaline pH (unpublished data), releasing sulfides that can be detected by subsequent incubation with NEM to form NEM-S-NEM. Thus, it will be possible to distinguish sulfide that is already present in the sample from sulfide derived from polysulfides by comparison of the values obtained from samples prepared by two different derivatization procedures, i.e., one is direct derivatization of sulfides at lower pH for detection of sulfide that is already present in the sample and the other one is decomposition of polysulfides to sulfides at higher pH in the presence of reductants and then derivatization of sulfides for detection of the whole sulfides, including polysulfides. However, it has been reported that alkylating agents can enhance alkaline hydrolysis of polysulfides [8,9,10], and thus, when NEM is utilized to derivatize sulfides, the results should be carefully considered. Combined with such procedures to distinguish derivatized sulfides, the anti-NEM-S-NEM rat mAb generated herein might be a useful tool for comprehensive measurement of polysulfides in biological samples, including cells, tissues, and various fluids.

## 5. Conclusions

In the present study, we employed NEM for derivatization of H_2_S, prepared NEM-S-MPA-conjugated protein as an antigen, and immunized rabbit and rat to generate polyclonal and monoclonal antibodies, respectively. Finally, we successfully generated two stable hybridoma cell lines (1C6 and 2D7) producing mAbs highly specific for NEM-S-NEM but not NEM-Cys and NEM. In fact, we determined H_2_S concentration in mouse plasma by competitive ELISA with the rat mAb developed herein, and the value was identical to that detected by LC-ESI-MS/MS analysis. However, presently, several important issues, such as time-consuming preparation steps for preparing biological samples and improvement of sensitivity and reproducibility, remain to be addressed. In future, in order to improve sensitivity, specificity, and reproducibility, and to more easily detect H_2_S in biological samples, conditions for pretreatment of biological samples, preparation of antigen proteins, and alternative detection methods, such as fluorescence and luminescence, need to be further optimized. To our knowledge, this is the first study to report antibody-mediated H_2_S detection. The mAb generated herein may be used to develop a convenient method to measure H_2_S levels as well as H_2_S pools (persulfides/polysulfides) in biological samples.

## Supplementary Materials

The following are available online at https://www.mdpi.com/2076-3921/9/11/1160/s1, Figure S1: Preparation of NEM-S-NEM, Figure S2: Preparation of NEM-labeled cysteine, Figure S3: Detection of NEM-S-MPA-conjugated peptide fragments of NEM-S-MPA-conjugated BSA protein by LC-ESI-MS, Figure S4: Detection of NEM-S-MPA-conjugated peptide fragments of NEM-S-MPA-conjugated OVA protein by LC-ESI-MS, Figure S5: In vitro assay for stoichiometrical detection of NEM-S-NEM by competitive ELISA, Table S1: Multiple reactions monitoring conditions by LC-ESI-MS/MS for detection of NEM-S-adducts, Table S2: Selected ion monitoring conditions by LC-ESI-MS for detection of NEM-S-MPA-containing peptide fragments produced by chymotrypsin digestion of NEM-S-MPA-conjugated BSA and OVA proteins., Table S3: The exact values in original mouse plasma determined by competitive ELISA and LC-ESI-MS/MS.
