# Supplementary material for: Generation of Rat Monoclonal Antibody to Detect Hydrogen Sulfide and Polysulfides in Biological Samples
Source: Antioxidants (Basel). 2020 Nov 21;9(11):1160. doi: 10.3390/antiox9111160 (PMC7700152; doi:10.3390/antiox9111160)
Supplement: Supplementary file 1 [file antioxidants-09-01160-s001.pdf]

Supplementary Information

Generation of rat monoclonal antibody to detect hydrogen sulfide and polysulfides in biological samples

Shingo Kasamatsu, Yuki Kakihana, Taisei Koga, Hisashi Yoshioka, and *Hideshi Ihara

Department of Biological Science, Graduate School of Science, Osaka Prefecture University, Osaka 599-8531, Japan.

*Corresponding author. Department of Biological Science, Graduate School of Science, Osaka Prefecture University, 1-1 Gakuen-cho, Sakai, Osaka 599-8531, Japan. Tel: +81-72-254-9753. Fax: +81-72-254-9163. E-mail address: ihara@b.s.osakafu-u.ac.jp (H. Ihara).

Supplementary Figure S1

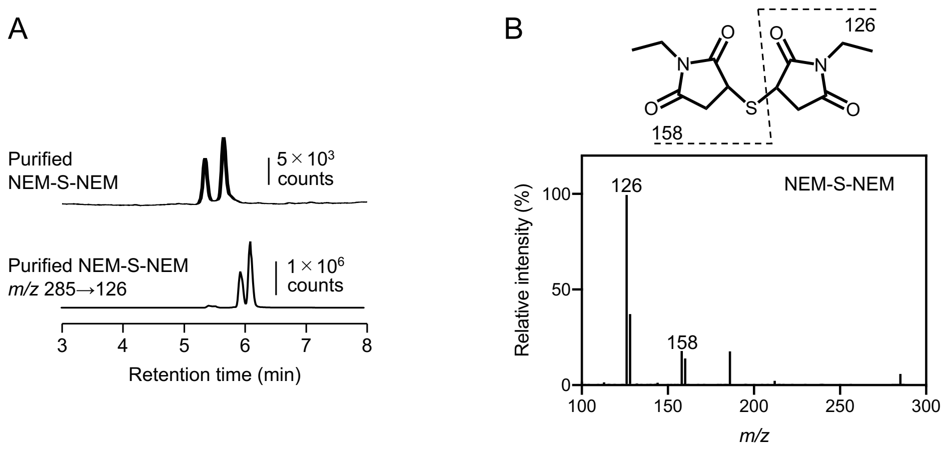

Supplementary Figure S1. Preparation of NEM-S-NEM. The detailed method of preparation and purification of bis-S-adduct of *N*-ethylmaleimide (NEM-S-NEM) was described in the section 2.2 of the main text. (A) Representative high-performance liquid chromatography (HPLC, *upper*) and liquid chromatography-electrospray ionization-tandem mass spectrometry (LC-ESI-MS/MS, *lower*) chromatograms of the purified NEM-S-NEM. (B) Mass spectra of fragment ions (*lower*) and assigned chemical structures (*upper*) indicating cleavage sites by dashed lines of purified NEM-S-NEM.

Supplementary Figure S2

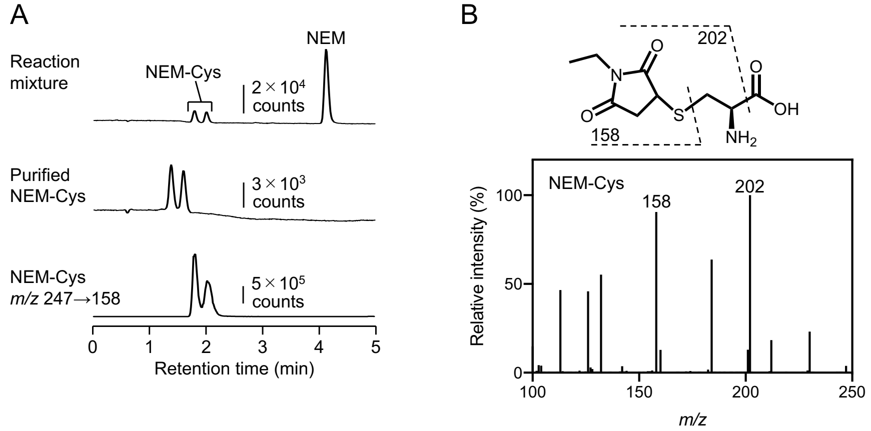

Supplementary Figure S2. Preparation of NEM-labeled cysteine. NEM-labeled cysteine (NEM-Cys) was prepared by a reaction of NEM and cysteine. (A) Representative HPLC chromatograms of the reaction mixture (*top*) and purified NEM-Cys (*middle*). Purified NEM-Cys was also confirmed by LC-ESI-MS/MS (*bottom*). (B) Mass spectra of fragment ions (*lower*) and assigned chemical structures (*upper*) indicating cleavage sites by dashed lines of purified NEM-Cys.

Supplementary Figure S3

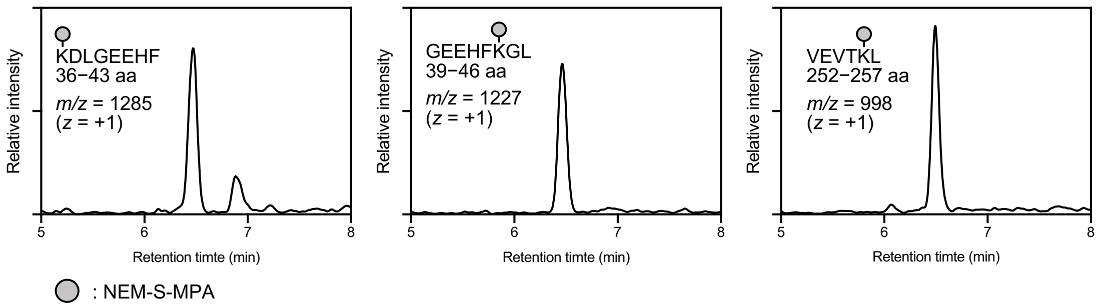

Supplementary Figure S3. Detection of NEM-S-MPA-conjugated peptide fragments of NEM-S-MPA-conjugated BSA protein by LC-ESI-MS. NEM-S-MPA-conjugated BSA protein was digested by chymotrypsin and the generated peptide fragments were analyzed by LC-ESI-MS analysis. Various peptide fragments containing NEM-S-MPA-adduct was detected by the mass spectrometry.

Supplementary Figure S4

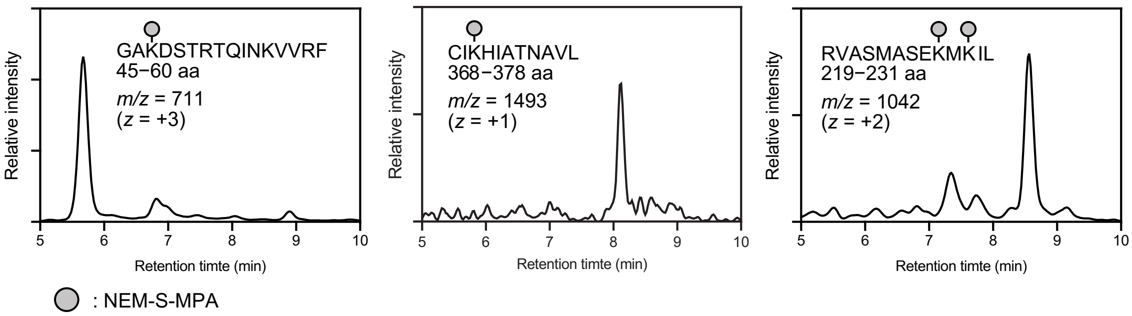

Supplementary Figure S4. Detection of NEM-S-MPA-conjugated peptide fragments of NEM-S-MPA-conjugated OVA protein by LC-ESI-MS. NEM-S-MPA-conjugated OVA protein was digested by chymotrypsin and the generated peptide fragments were analyzed by LC-ESI-MS analysis. Various peptide fragments containing NEM-S-MPA-adduct was detected by the mass spectrometry.

Supplementary Figure S5

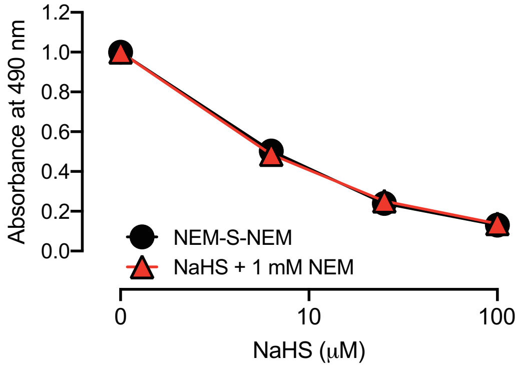

Supplementary Figure S5. *In vitro* assay for stoichiometrical detection of NEM-S-NEM by competitive ELISA. NEM (1 mM) was incubated with various concentrations of NaHS (6.3, 25, 100 μM) at 37˚C for 1 h, and the formed NEM-S-NEM in the reaction mixture was detected by competitive ELISA with anti-NEM-S-NEM mAb (clone 1C6). Purified NEM-S-NEM was used as a standard.

Supplementary Table S1

Analyte	Precursor ion (*m/z*)	Product ion (*m/z*)	Collision energy (V)
NEM-S-MPA	329	257	20
NEM-S-NEM	285	126	20
NEM-Cys	247	158	20

Multiple reactions monitoring conditions by LC-ESI-MS/MS for detection of NEM-S-adducts. NEM-S-MPA: bis-S-heteroadduct with NEM and 3-maleimidopropionic acid, NEM-S-NEM: bis-S-adduct of NEM; NEM-Cys: NEM-labeled cysteine.

Supplementary Table S2

Protein	Amino acid sequence	Position	Precursor ion (m/z)	Charge	Cone voltage (V)
NEM-S-MPA -conjugated BSA	AKY	284−286 aa	691.0	+1	35
	VEVTKL	252−257 aa	998.0	+1	35
	EKLGEY	419−424 aa	1048.0	+1	35
	GEEHFKGL	39−46 aa	1227.0	+1	35
	KDLGEEHF	36−43 aa	1285.0	+1	35
NEM-S-MPA -conjugated OVA	GAKDSTRTQINKVVRF	45−60 aa	711.0	+3	35
	LGAKDSTRTQINKVVRF	44−60 aa	748.0	+3	35
	LPRMKMEEKYNL	283−294 aa	931.0	+2	35
	RVASMASEKMKIL	219−231 aa	1042.0	+2	35
	CIKHIATNAVL	368−378 aa	1493.0	+1	35

Selected ion monitoring conditions by LC-ESI-MS for detection of NEM-S-MPA-containing peptide fragments produced by chymotrypsin digestion of NEM-S-MPA-conjugated BSA and OVA proteins.

Supplementary Table S3

Method	Mean ± SE
Competitive ELISA	0.18 ± 0.008 μM
LC-ESI-MS/MS	0.20 ± 0.004 μM

The exact values in original mouse plasma determined by competitive ELISA and LC-ESI-MS/MS. SE, standard error.
